# Malignant Transformation in Leukoplakia and Its Associated Factors in Southern Iran: A Hospital Based Experience

**Published:** 2017-08

**Authors:** Alireza BARFI QASRDASHTI, Mina Seied HABASHI, Peyman ARASTEH, Mahshid TORABI ARDAKANI, Zahra ABDOLI, Seyed Sajjad EGHBALI

**Affiliations:** 1. Dept. of Prosthodontics, Shiraz University of Medical Sciences, Shiraz, Iran; 2. Dept. of Endodontics, Shiraz University of Medical Sciences, Shiraz, Iran; 3. Noncommunicable Diseases Research Center, Fasa University of Medical Sciences, Fasa, Iran; 4. MPH Dept., Shiraz University of Medical Sciences, Shiraz, Iran; 5. Biomaterial Research Center, Shiraz University of Medical Sciences, Shiraz, Iran; 6. Dept. of Pediatric Dentistry, School of Dentistry, Shiraz University of Medical Sciences, Shiraz, Iran; 7. Dept. of Pathology and Lab Medicine, Bushehr University of Medical Sciences, Bushehr, Iran

**Keywords:** Oral lesion, Leukoplakia, Malignant transformation, Iran

## Abstract

**Background:**

We evaluated factors that affect malignant transformation of leukoplakia in a sample of the Iranian population.

**Methods:**

The records of patients with a clinical diagnosis of leukoplakia during a 20-year period from 1989–2009 referred to two of the largest referral centers in southern Iran were studied. Patients that developed malignant transformation were compared with patients that did not have malignant changes.

**Results:**

Of 522 patients, female patients, those over 50 yr old and with lesions located on the tongue had the highest rate of malignant changes. Female patients with malignant changes were mostly non-smokers (76.4%), while male patients with malignant changes were mostly smokers (63.8% in non-smokers) (*P*<0.001). In our univariate analysis, male sex and smoking showed lower chances for malignant transformation (OR: 0.57; CI=0.397–0.822 and OR: 0.025; CI=0.141–0.299, respectively), while age above 50 was a risk factor for malignant transformation (OR: 3.57; CI=2.32–5.42). In the multivariate analysis, smoking (OR: 0.317; 95% CI=0.16–0.626) and morphological presentation as erythroplakia (OR: 0.025; 95% CI=0.005–0.131) had low chances for developing malignant changes, while site of lesion on the tongue (OR: 774; 95% CI=60–9838) and morphological presentation as erythroleukoplakia (OR: 6.26; 95% CI=3.16–12.38) were a risk factor for developing malignant changes

**Conclusion:**

A follow-up program and further work-up should be considered for Iranian patients who have a leukoplakia lesion that is flat and are white patch or plaques with red components, in addition for patients who have lesions located on the tongue and for nonsmokers who develops leukoplakia lesions.

## Introduction

Oral cancers are the sixth most common type of cancer among males and the twelfth most common type of cancer among females in the US. The most prevalent type of pre-cancerous ulcer in the oral cavity is leukoplakia, constituting about 85% of all oral lesions with an estimated global prevalence of 2.60% (1, 2).

Based on the definition by the World Health Organization (WHO), leukoplakia is considered a white plaque or patch which does not resemble any other ulcer pathologically or clinically. All leukoplakia lesions do not necessarily transform to malignancies and some show signs of complete withdrawal and even complete cure, however, the lesion itself is considered as potentially malignant (3, 4). Retrospective studies have documented different results regarding the rate of malignant transformation in leukoplakia lesions ranging from 0.13% to 36.4% (2, 5).

Multiple factors have been shown to play a role in the malignant transformation of a leukoplakia lesion including patients’ habits, genetic factors, existence of dysplasia and/or candidiasis infections, and age (6–8). The etiology and the occurrence of dysplastic change in leukoplakia are still not completely understood, furthermore cases of malignant transformation of hyperkeratosis leukoplakia without dysplastic change have been reported (9).

Studies have shown different and in some cases, contradicting results regarding the risk factors that contribute to the malignant transformation of these lesions based on geographical region, cultural and lifestyle differences, which include a wide range of environmental factors and dietary habits (5, 10).

In order to understand region-specific risk factors for malignant change in leukoplakia lesions, in this study we evaluated the factors that affect malignant transformation in a sample of the Iranian population diagnosed with leukoplakia.

## Materials and Methods

### Study settings and protocol

This study was conducted in Namazi and Khalili hospitals (Shiraz, Iran), which are two of the largest referral centers in southern Iran.

The study protocol was approved by the Institutional Review Board of Shiraz University of Medical Sciences. Guidelines of the Declaration of Helsinki were followed in all stages of the study. In this retrospective study, the records of all patients that referred for biopsy with a clinical diagnosis of leukoplakia during a 20-year period from Jan 1989 to Dec 2009, were considered for inclusion in the study. Overall, 613 patients were initially considered for inclusion as the study population. Patients’ secrecy of data was maintained throughout the study and no personal information was disclosed regarding any of the patients.

Patients that developed malignant changes in their leukoplakia lesions were considered as group 2 and those who did not develop any malignant change were considered as the benign group or group 1. Any patient that had missing data, had any other type of leukoplakia (other than oral) or had any other systemic or infectious disease was excluded from the study.

Patients’ records were reviewed and information regarding age, sex, site of lesion, clinical morphology and smoking were registered and evaluated.

Our patient information sheet was made of two different parts. The first part included demographic information that was obtained from the records and the second part included information on personal habits like smoking. For obtaining information regarding personal habits, we contacted and visited the patients and asked them about their history of smoking and their amount of tobacco use.

### Measurements

The site of lesion was classified into one of the following locations: gingiva of the upper jaw, gingiva of the lower jaw, mucosa of the buccal area, tongue, lips and the base of the mouth.

Regarding clinical morphology, we classified the lesions into four categories: type 1 as flat and white patches or plaques without red components, type 2 as flat and white patch or plaques with red components (erythroleukoplakia), type 3 as red patch or plaques (erythroplakia) and type 4 as raised or elevated white plaques.

Patients were classified as having malignant lesions according to their biopsy results.

For better evaluation of patients based on their age, we classified them into 8 age groups of <19, 20–29, 30–39, 40–49, 50–59, 60–69, 70–79 and 80–89 yr old. In another classification, patients were also classified as above 50 yr old and bellow 50 yr old.

### Statistical analysis

The study population size was defined using a pilot study and considering a power of 80% and α of 5%, accordingly an estimated 210 people were needed in each group. Data were analyzed using the SPSS for Windows, ver. 18 (SPSS Inc., Chicago, IL, USA). For comparison of quantitative data between two groups, the independent t-test was used and for comparison of qualitative data, the chi-square test was used. We used a univariate analysis to show risk factors for developing malignant change, reporting its odds ratio (OR) and 95% confidence interval (CI). We used a multivariate analysis using a logistic regression model to evaluate the simultaneous effect of different factors for the development of malignant changes. A two-tailed *P*-value of less than 0.05 was considered as statistically significant.

## Results

After reviewing for missing data, 522 cases entered the study. Among these 522 cases, 210 were males. Overall, 213 patients showed signs of malignant transformation (group 2). Patients younger than 50 yr old mostly had benign lesions (78%), however, patients over 50 yr old mostly had lesions that developed malignant change (50.2%) (*P*<0.001).

The two groups showed a significant difference regarding their site of lesion (*P*<0.001). Majority of the lesions located on the tongue (63%) developed malignant change. This was the same for lesions located on the base of the mouth (54%). Erythroplakia lesions were mostly benign (93.3%), while flat and white patches or plaques without red components, erythroleukoplakia and raised or elevated white plaques mostly underwent malignant changes (71%, 71%, and 56.7%, respectively) (*P*<0.001).

Most smokers had benign lesions (74.5%), while most non-smokers developed malignant lesions (62.5%) (Table 1).

**Table 1:** Patients' baseline and leukoplakia related characteristics in the two groups^*^

**Variables**	**Group 1 (n=309)**	**Group 2 (n=213)**		
**Sex**	**Frequency – no. (%)**	**Frequency – no. (%)**	**Total**	***P*-value**
Male	141 (67.1)	69 (32.9)	210	
Female	168 (53.8)	144 (46.2)	312	0.002
**Age**				
< 50	135 (78)	38 (22)	173	
> 50	174 (49.8)	175 (50.2)	349	<0.001
**Site of lesion**				
Upper gingiva	38 (86)	6 (14)	44	
Lower gingiva	36 (78)	10 (22)	46	
Buccal	96 (67.6)	46 (32.4)	142	<0.001
Tongue	65 (36.1)	115 (63.9)	180	
Lips	48 (89)	6 (11)	54	
Base of mouth	26 (46)	30 (54)	56	
**Clinical morphology†**				
1	24 (29)	58 (71)	82	
2	23 (29)	56 (71)	79	<0.001
3	197 (93.3)	14 (6.7)	211	
4	65 (43.3)	85 (56.7)	150	
**Smoking**				
Yes	228 (74.5)	78 (25.5)	306	<0.001
no	81 (37.5)	135 (62.5)	216	

* Group 1 represents patients with leukoplakia without malignant transformation and group 2 represents those with malignant transformation. Some of the numbers have been rounded for better evaluation.

† Clinical morphology was considered as type 1: flat and white patches or plaques without red components; type 2: flat and white patch or plaques with red components (erythroleukoplakia); type 3: red patch or plaques; type 4: raised or elevated white plaques.

We compared males and females in the two groups based on different variables. Our results showed that in group 2, males and females had a significant difference in their distribution of age groups (*P*<0.001). In group 1, most of the patients (both male and females) were between the ages of 50–59 yr old. In group 2, females were mostly between 60–69 yr old, while male patients were between 50–59 yr old. In females, majority of malignant transformations were related to those lesions located on the tongue, while in males both the tongue and the buccal area were common areas for malignant transformation. The difference between males and females was significant regarding the location of leukoplakia (*P*<0.001). Although for benign lesions males and females displayed significant differences in clinical morphology of leukoplakia (*P*<0.001), in group 2, there was no difference between the two sexes (*P*=0.4). Female patients with malignant changes were mostly non-smokers (76.4%), while males in the group with malignant changes were mostly smokers (63.8% in non-smokers) (*P*<0.001) (Table 2).

**Table 2:** Comparison of variables between males and females among the two group^*^

**Variables**	**Group 1** (**n=309)**			**Group 2 (n=213)**		
	**Frequency – no. (%)**		***P*-value**	**Frequency – no. (%)**		***P*-value**
	**Male (n=141)**	**Female (n=168)**		**Male (n=69)**	**Female (n=144)**	
**Age†**						
< 19	2 (1.4)	0		0	0	
20 – 29	20 (14.1)	0		4 (5.7)	0	
30 – 39	19 (13.4)	11 (6.5)		5 (7.2)	4 (2.7)	
40 – 49	32 (22.6)	51 (30.3)	0.125	9 (13)	16 (11.1)	<0.001
50 – 59	48 (34)	57 (33.9)		32 (46.3)	37 (25.6)	
60 – 69	12 (8.5)	24 (14.2)		11 (15.9)	62 (43)	
70 – 79	7 (4.9)	24 (14.2)		7 (10.1)	22 (15.2)	
80 – 89	1 (0.7)	1 (0.5)		1 (1.4)	3 (2)	
						
< 50	73 (51.8)	62 (36.9)		18 (26.1)	20 (13.9)	
> 50	68 (48.2)	106 (63.1)	0.009	51 (73.9)	124 (86.1)	0.03
**Site of leukoplakia**						
Upper gingiva	7 (5)	31 (18.5)		2 (2.9)	4 (2.8)	
Lower gingiva	9 (6.4)	27 (16.1)		4 (5.8)	6 (4.2)	
Buccal	27 (19.1)	69 (41.1)		23 (33.3)	23 (16)	
Tongue	54 (38.2)	11 (5.5)	0.125	27 (39.1)	88 (61.1)	<0.001
Lips	34 (24.1)	14 (8.3)		4 (5.8)	2 (1.4)	
Base of mouth	10 (7.2)	16 (9.5)		9 (13)	21 (14.6)	
**Clinical morphology‡**						
1	13 (9.2)	11 (6.5)		15 (21.7)	43 (29.9)	
2	21 (14.9)	2 (1.2)		21 (30.4)	35 (24.3)	
3	85 (60.3)	112 (66.7)	<0.001	21.4 (4.3)	11 (7.6)	0.4
4	22 (15.6)	43 (25.6)		30 (43.5)	55 (38.2)	
**Smoking**						
Yes	119 (84.4)	109 (64.9)	<0.001	44 (63.8)	34 (23.6)	<0.001
no	22 (15.6)	59 (35.1)		25 (36.2)	110 (76.4)	

In our univariate analysis, male patients had a lower chance for developing malignant transformation (OR: 0.57; 95% Cl = 0.397–0.822). This was the same with smoking (OR: 0.205; 95% Cl: 0.141–0.299). Age over 50 yr old was a risk factor for malignant change (OR: 3.57; 95% Cl = 2.35 – 5.42) (Table 3).

**Table 3:** Odds ratio of different variables regarding malignant transformation

**Variables**	**Odds ratio**	**95% Confidence interval**
**Sex**		
Male	0.57	0.397 – 0.822
**Age – yr**		
> 50	3.57	2.35 – 5.42
**Smoking**		
Yes	0.205	0.141 – 0.299

In our multivariate regression model, the effects of sex and age on malignant changes were abrogated. Smoking (OR: 0.317; 95% CI = 0.16–0.626) and morphological presentation as erythroplakia (OR: 0.025; 95% CI = 0.005–0.131) had low chances for developing malignant changes, while site of lesion on the tongue (OR: 774; 95% CI = 60–9838) and morphological presentation as erythroleukoplakia (OR: 6.26; 95% CI = 3.16–12.38) were a risk factor for developing malignant changes (Table 4).

**Table 4:** Logistic regression for evaluating risk factors for malignant transformation

**Variables**	**Odds ratio**	**95% Confidence interval**
**Sex**		
Male	1.08	0.555 – 2.128
**Age – yr**		
> 50	1.8	0.877 – 3.69
**Smoking**		
Yes	0.317	0.16 – 0.626
Clinical morphology^*^		
Type 2	6.26	3.164 – 12.38
Type 3	0.025	0.005 – 0.131
Type 4	0.983	0.359 – 2.69
**Site of lesion†**		
Tongue	774.556	60.94 – 9838.64
Lower gingiva	0.513	0.115 – 2.27
Buccal	0.626	0.117 – 2.21
Lips	0.293	0.058 – 1.47
Base of mouth	0.533	0.142 – 2.004

* Type 1 clinical leukoplakia or flat, white patches or plaques without red components were considered as the base for comparison in the regression model.

Type 2: flat and white patch or plaques with red components (erythroleukoplakia); type 3: red patch or plaques; type 4: raised or elevated white plaques.

† For the site of leukoplakia, the upper gingiva was considered as the base for comparison in the regression model.

## Discussion

The goal of our study was to evaluate regional factors that contribute to malignant changes in leukoplakia lesions in the Iranian population. We found that females and those over 50 yr old had more leukoplakia with malignant transformation. The most common site for malignant changes was on the tongue. In our multivariate analysis, erythroleukoplakia and location of a leukoplakia lesion on the tongue were risk factors for malignant change, while erythroplakia lesions and leukoplakia associated with smoking were unlikely to develop malignant changes.

In a cohort study from Amsterdam including 144 patients (11), who had a definitive diagnosis of leukoplakia, malignant transformation rate was 11% (vs. 40.8% in our study). Female patients with leukoplakia lesions were more prevalent and higher rates of malignant transformation were seen in females, however, this difference was not statistically significant (*P*=0.609). This was similar to our findings, although we found the difference between the sex groups to be significant regarding malignant transformation. Moreover, the tongue was found to be the most common site for malignant change, however unlike our study they did not document a significant relation between location of lesion and malignant transformation (*P*=0.144). These statistically insignificant findings may be attributed to their relatively low population size, which may not have been able to show a meaningful difference between the comparison groups.

Malignant changes in non-homogenous lesions was 7 times higher compared to homogenous lesions (OR=7), which was similar to our result regarding the higher risk of malignant changes in erythroleukoplakia (type 2) compared to homogenous lesions (type 1) (OR= 6.26; 95% CI: 3.164 – 12.38) (12).

In a large retrospective study in Croatia (2), similar to our results, females and smokers made up the majority of patient with leukoplakia (57% and 47%, respectively), which was statistically significant (*P*<0.001). The higher frequency of leukoplakia in the female sex may seem contradicting as males have higher rates of smoking compared to females (13). The reason for this may be due to multiple factors. First, in our study population, the self-reported smoking rate in Iranian women is less than the actual number of smokers (14), although minor, this may have affected the results. Second is the fact that females seek medical attention more frequently than males for lesions like leukoplakia, which increased their number compared to males.

In the same study (2), their malignant transformation rate was extremely lower than that documented in our study (0.64% vs. 40.8%), however majority of their patients had homogenous lesions, unlike our patients who had nonhomogenous lesions, furthermore our study had a much longer follow-up period.

Our findings suggested that flat and white patch or plaques with red components were mostly associated with malignant change. This was also reported earlier where erythroleukoplakia had the highest chances for malignant transformation (15). Almost every study up to this date has found that a non-homogenous morphology for leukoplakia is associated with carcinomatous change (15). We found that as lesions change from a flat and homogenous structure to a more erythematous and complex structure, it is more likely to undergo malignant changes. In one study, the risk of malignancy in erythroleukoplakia was 4 times higher than that of a homogenous leukoplakia and among those that underwent malignant changes, 84% had red structures (9).

Malignant changes were evaluated among patients with proliferative verrucous leukoplakia, which is an aggressive form of leukoplakia. In this study, cancer developed more frequently in non-smokers and females (up to four times) (16). Non-smokers also had higher prevalence of verrucous leukoplakia; this supports our findings that non-smokers acquire more aggressive forms of leukoplakia. Patients also had a mean age of 61.7 yr old showing that older patients acquire more severe forms of leukoplakia. The most common locations for this type of lesion were in the gingiva and on the tongue, respectively. In another study similar to the later study (17), only age and site of verrucous leukoplakia contribute to malignant changes in these types of lesions. The tongue and buccal mucosa were the most common sites for verrucous leukoplakia.

The reason for our high rates of malignant changes when compared to studies from other countries, could be related to cultural differences, for example, many of Iranians who have leukoplakia lesions only refer for medical evaluation when the lesion has advanced, consequently showing high rates of malignant changes in biopsy reports. Another factor is that our study was conducted in two referral hospitals (for southern and central Iran) where most patients who are referred are medically complicated.

Our results showed a significant difference in malignant transformation rates between patient bellow 50 yr old and those over 50 yr old. These findings were also supported earlier (15). This finding might be because older patients may have had the leukoplakia for a longer amount of time, giving the lesion time to undergo malignant transformation.

Data on the prevalence of malignant lesions and malignant changes in the two sexes have been somewhat contradicting in previous literature. Female patients have had higher rates of malignant changes in some reports (18, 19), while others have shown that these changes are more prevalent in males (20).

We found a higher prevalence of leukoplakia among smokers. However, lower rates of malignant changes were found in smokers. One explanation for these phenomena might be that in smokers, tobacco is the cause of leukoplakia meanwhile in non-smokers other more dangerous carcinogenic factors might be the cause of leukoplakia, thus having higher chances of malignant changes.

The site of malignant change is probably related to the habits of the patients (21), for example, patients who chew tobacco are prone to acquiring malignant transformation in the buccal area and lips. The most common site for malignant changes in smokers was on the base of the mouth, while in non-smokers was on the side of the tongue (22). Majority of the women in our study were non-smokers; furthermore, they constituted the majority of our patients with malignant changes. This could have been the cause for the tongue as to being the most common site for malignancy. Overall, the different results among studies are due to different follow-up periods, different settings of study and the treatment that each patient received.

Our study had some limitations. It was not feasible for us to consider genetic variation among patients as one study indicated that this could have affected malignant transformation (23). The retrospective nature of our study did not allow us to document a causality relationship between malignant change and different factors; furthermore, it did not allow us to have a single observer for defining the clinical morphology of the lesions, which may have affected the classification of some lesions.

This is the first study to evaluate common and region-specific risk factors for malignant changes of leukoplakia in the Iranian population with an appropriate sample size. We also had one of the longest follow-ups among all the studies that have considered malignant changes in leukoplakia. Cohort studies with long follow-ups are needed to better clarify the causes and risk factors of malignant transformation in patients with leukoplakia.

## Conclusion

A follow-up program and further work up should be considered for Iranian patients who have leukoplakia lesions that are flat and are white patch or plaques with red components, and for patients who have lesions located on the tongue and for nonsmokers who develops leukoplakia lesions.

## Ethical considerations

Ethical issues (Including plagiarism, informed consent, misconduct, data fabrication and/or falsification, double publication and/or submission, redundancy, etc.) have been completely observed by the authors.
